# Supplementary material for: Vitamin B Mitigates Thoracic Aortic Dilation in Marfan Syndrome Mice by Restoring the Canonical TGF-β Pathway
Source: Int J Mol Sci. 2021 Oct 29;22(21):11737. doi: 10.3390/ijms222111737 (PMC8583889; doi:10.3390/ijms222111737)
Supplement: Supplementary file 1 [file ijms-22-11737-s001.zip › Supplementary Materials.pdf]

Supplementary Materials:

Echocardiography

Mouse thoracic aortas were examined by echocardiography at the age of 24 weeks. Echocardiography was performed by Taiwan Mouse Clinic. Mice were anesthetized by the inhalation of isoflurane, and chest hair was removed. The parasternal long-axis view was examined by ultrasound (Vevo3100). The aortic root diameter was measured at diastolic intervals.

Paraffin Cross-Section and Verhoeff–Van Gieson Staining

The orientation of the aortic root significantly affects the outcomes of histological analysis. We embedded aortic tissue in a 4% agarose gel before paraffin embedding. Three-micrometer paraffin cross-sections were examined slide-by-slide during sectioning to correct the vertical orientation of aortic tissues. We precisely determined the position of the sinotubular junction (STJ) and confirmed the correct size of each section. Each slide contained three cross-sections with 9 µm intervals at the STJ section for further analysis. Modified Verhoeff-van Gieson (VVG) staining reagents (26369-01~05, Electron Microscopy Science, Hatfield, PA, USA) were used to stain the elastic fibers in aortic tissues. The lengths of the internal and external elastic lamina (EEL) were measured by ImageJ software. The aortic diameter and aortic media thickness were calculated with formulas.

Tail-Cuff Pulse Rate Experiment

Blood pressure experiments were performed by the Taiwan Mouse Clinic when mice were 24 weeks old. Mice were transferred into a quiet and independent room for the experiment. Using a BP-2000 Series II Blood Pressure Analysis System, blood pressure was measured from mouse tails without anesthesia or any invasive methods.
