# Supplementary material for: Vitamin B Mitigates Thoracic Aortic Dilation in Marfan Syndrome Mice by Restoring the Canonical TGF-β Pathway
Source: Int J Mol Sci. 2021 Oct 29;22(21):11737. doi: 10.3390/ijms222111737 (PMC8583889; doi:10.3390/ijms222111737)
Supplement: Supplementary file 1 [file ijms-22-11737-s001.zip › supplemetal figures_2021.10.18.pdf]

Supplemental Figure S1

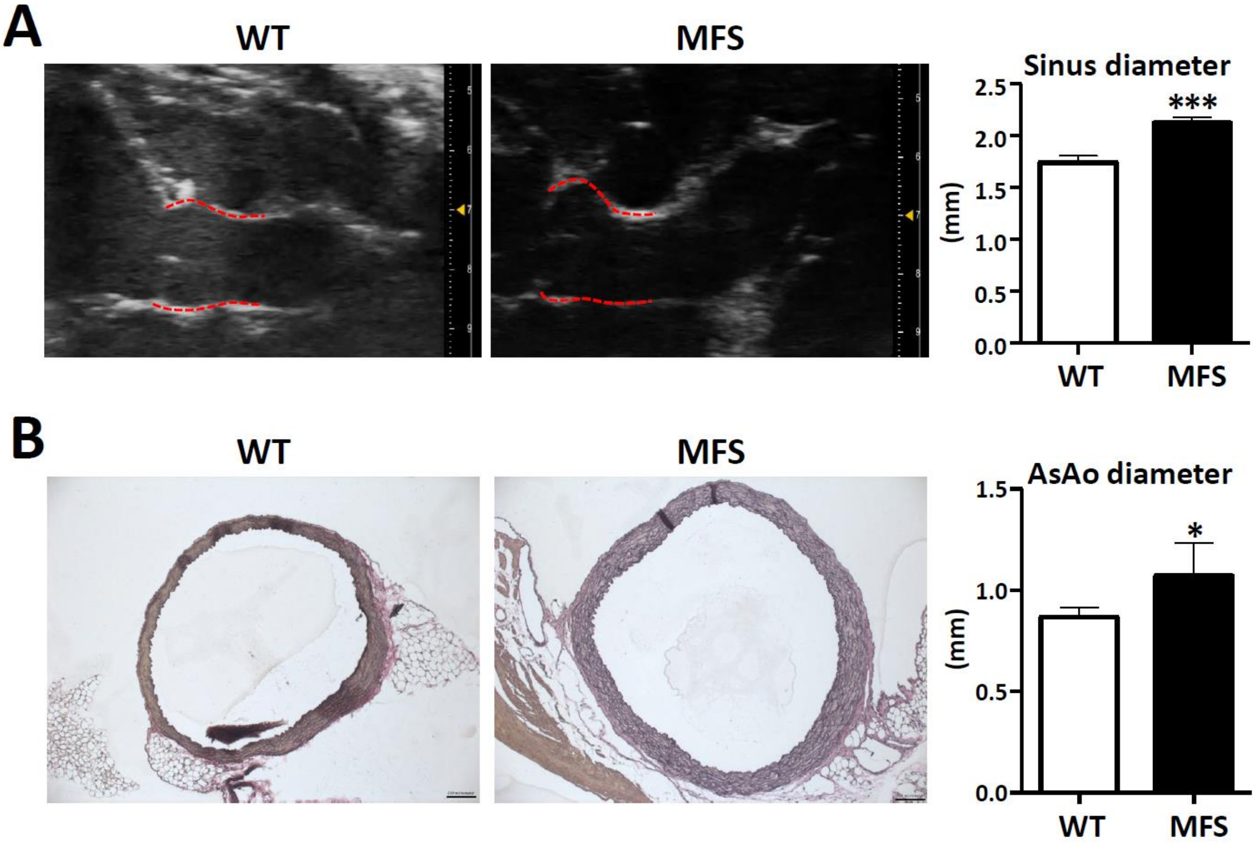

Supplemental Figure S1. Thoracic aortic aneurysm of Marfan syndrome mouse model at the age of 6 months. (A) Representative aortic root echocardiograph (parasternal long-axis view) and quantification of Sinus diameter (WT, n=3; MFS, n=4). **(B)** Representative Verhoeff-Van Gieson stain of ascending aorta (AsAo) and quantification of AsAo diameter (n=6 per group). Scale bar, 100μm. Data are presented as Mean ± SD. **p*<0.05, ****p*<0.001.

Supplemental Figure S2

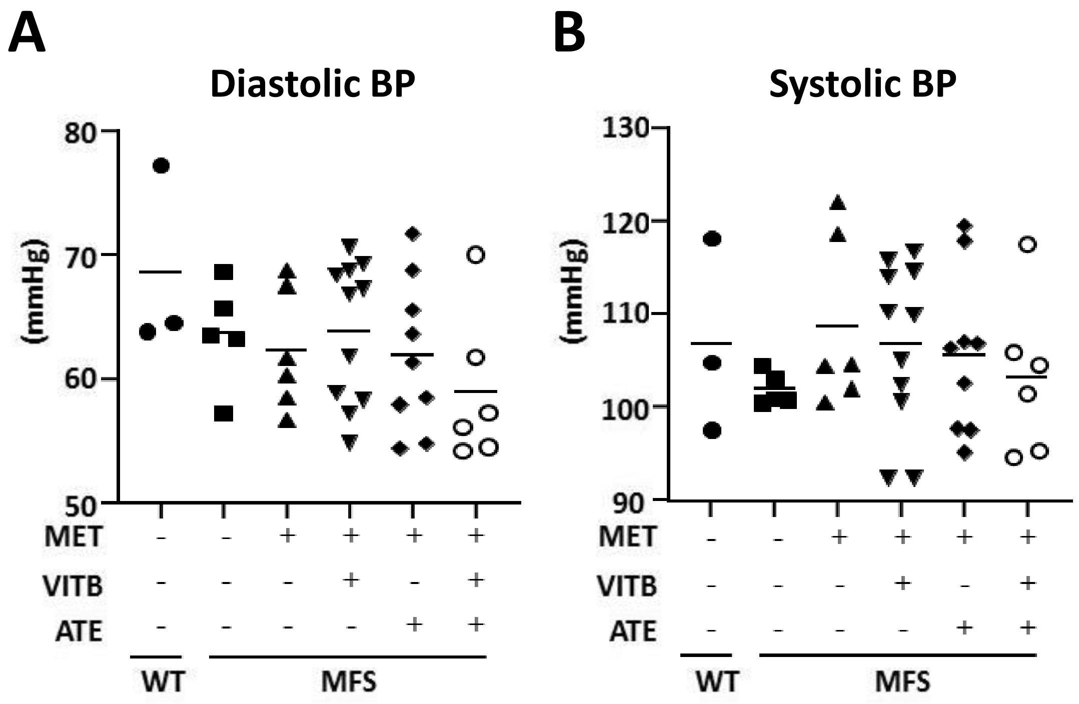

Supplemental Figure S2. The effect of methionine and VITB treatment on blood pressure. (A) Diastolic blood pressure. **(B)** Systolic blood pressure. No significant difference was found in blood pressure between treatment groups. WT, wild-type; C1039G/+, Marfan syndrome mouse model; MET, methionine in drinking water; VITB, vitamin B complex treatment; ATE, Atenolol treatment.
